# Left Ventricular Pseudoaneurysm Following Penetrating Chest Trauma: A Case Report

**DOI:** 10.7759/cureus.53749

**Published:** 2024-02-07

**Authors:** Mohammad Abu-Baker, Amro AlAqra, Nadine Yaghi

**Affiliations:** 1 Cardiac Surgery, An-Najah National University Hospital, An-Najah National University, Nablus, PSE; 2 General Surgery, Faculty of Medicine, Al-Quds University, Jerusalem, PSE

**Keywords:** left ventricular pseudoaneurysm, sutures, stab, wounds, false, aneurysm

## Abstract

Post-traumatic ventricular pseudoaneurysms are a rare complication of chest trauma that necessitate surgical correction. In this case report, we describe a 22-year-old male patient presenting with a left ventricular pseudoaneurysm 45 days following primary surgical repair of a penetrating left ventricular injury with a background of stabbing chest trauma. The pseudoaneurysm was successfully surgically treated at our hospital after a thorough evaluation despite the vague clinical presentation at the time of referral. The patient fully recovered afterward and his case enriched our understanding of this life-threatening yet rare complication.

## Introduction

Left ventricular pseudoaneurysms, also referred to as false aneurysms, are a rare but potentially fatal complication with high mortality [1]. This condition can be caused by various etiologies, most commonly following myocardial infarction, previous cardiac surgery, infections, or, less frequently, chest wall trauma [1]. The mechanism by which pseudoaneurysm forms is cardiac wall rupture that becomes contained within the adherent pericardium or surrounding scar tissue and expands over time. Unlike true aneurysms, the wall of a pseudoaneurysm lacks myocardium and endocardium with a narrow neck compared to the diameter of the sac [2]. Their high tendency to rupture makes them extremely dangerous, and their poor prognosis requires early recognition and management with emergency surgery. If left untreated, spontaneous rupture may lead to cardiac tamponade, shock, and death, in contrast to the more indolent course of a benign true aneurysm [3].

Here, we present a unique case of a 22-year-old male patient who developed a left ventricular pseudoaneurysm 45 days after the primary repair of a penetrating stab wound injury to the left ventricle.

## Case presentation

A 22-year-old male patient, with a past surgical history of left ventricular stab wound injury addressed by primary repair through a left thoracotomy two months before presentation, was admitted to the cardiothoracic surgery department of our hospital (tertiary center) as a case of left ventricular pseudoaneurysm.

**Figure 1:**
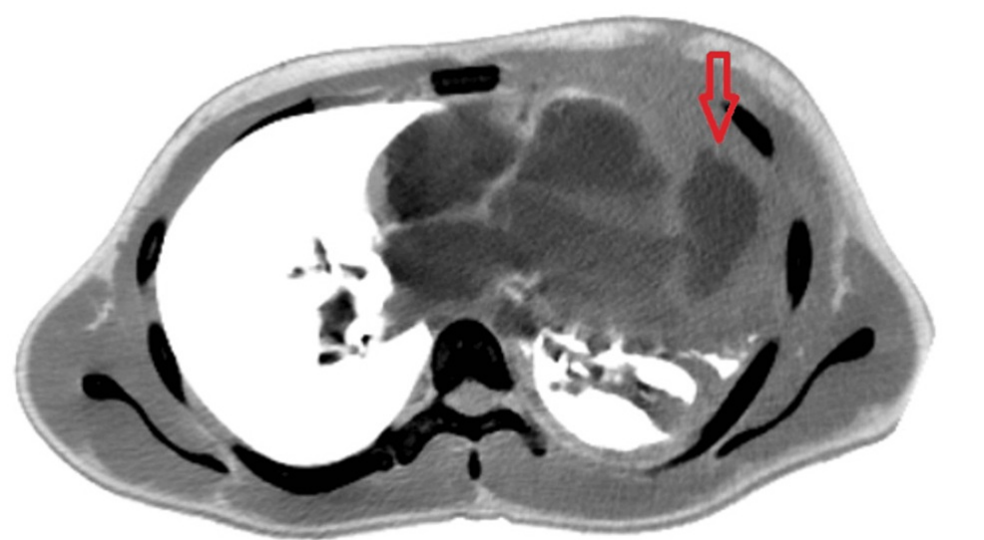
Chest CT scan showing the pseudoaneurysm (pointed by the red arrow) communicating with the left ventricular cavity through a narrow neck.

Forty-five days following chest wall trauma he started to complain of shortness of breath accompanied by high-grade fever. A chest CT scan with intravenous contrast showed left lung consolidation, in addition to an incidental finding of a cavity-like structure communicating with the left ventricle consistent with a pseudoaneurysm (Figure 1). Hence, the patient was referred to our cardiac surgery department for further evaluation and possible surgical resection of the ventricular pseudoaneurysm.

**Figure 2:**
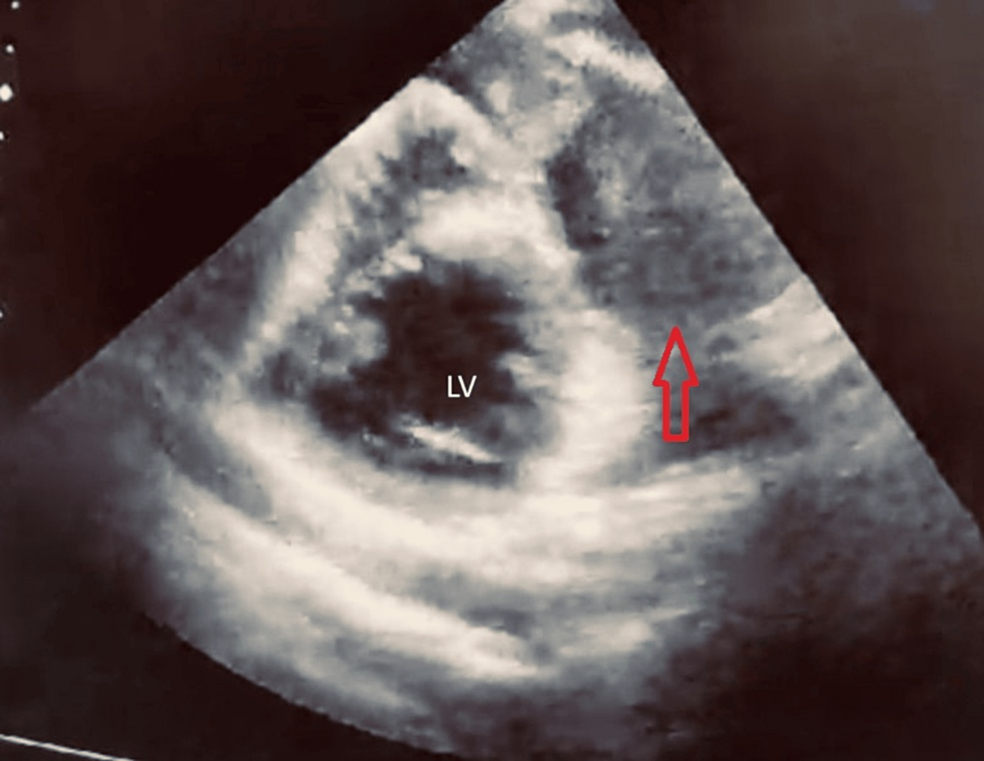
Transthoracic echocardiogram showing the pseudoaneurysm cavity (pointed by the red arrow) communicating with the left ventricular cavity.

Upon arrival at our emergency department, the patient was hypotensive with a blood pressure of 70/33 mmHg, tachycardic at 130 beats/minute, with oxygen saturation of 77% on room air, and a temperature of 39°C. His ECG showed no specific findings. Resuscitation with intravenous fluids and oxygen therapy resulted in an improvement in his hemodynamic status. After stabilization, a transthoracic echocardiography was conducted in the emergency department confirming the presence of a large pseudoaneurysm with a valve-like structure around the apex of the left ventricle, with a bidirectional flow (Figure 2).

In a multidisciplinary team, it was concluded that the patient requires resection of the pseudoaneurysm after stabilization of his medical condition, as surgery in the presence of ongoing sepsis (on the background of pneumonia in our case) carries a high mortality risk.

After 10 days of medical treatment with appropriate antibiotics according to culture sensitivity, the patient’s condition ameliorated, and he was declared by our multidisciplinary team fit for surgery.

The patient underwent surgical resection of the pseudoaneurysm and closure of the communicating defect by direct suture. A median sternotomy was performed, and purse string sutures over the ascending aorta and the right atrium were secured for any emergency. We observed extensive adhesions between the left ventricle and the left chest wall anteriorly and to the pericardium posteriorly. The left lung was not completely identified as it was atelectatic and adherent to the pseudoaneurysm as one unit. Dissection of the adhesions was carefully done with no immediate complications, separating the left ventricle from the chest wall till the exposure of the pseudoaneurysm. Total cardiopulmonary bypass was instituted through an arterial cannula in the ascending aorta and a double-staged venous cannula in the right atrium, and the patient was cooled down to 34°C. The pseudoaneurysm was then opened with findings of a huge thrombus situated within it which was evacuated. The communication orifice between the left ventricle and the pseudoaneurysm was identified (2.5 cm in length) and was closed with pledgeted Teflon.

The postoperative period was uneventful without any complications. The patient was kept under supervision for five days before being discharged home in good medical condition. Two weeks following his discharge he was seen at our cardiac surgery clinic, where he reported that he returned to his normal lifestyle without any limitations and had no further complaints.

## Discussion

Post-traumatic left ventricular pseudoaneurysms are rare but potentially fatal complications in the cardiac surgery field. Although most cases are caused by iatrogenic injuries, the literature describes a few cases of blunt and more commonly penetrating trauma leading to ventricular pseudoaneurysms [4]. The symptoms if present include congestive heart failure, angina, dyspnea, and sudden cardiac death, with a 30-45% risk of rupture if left untreated as they tend to grow rapidly [5]. Unfortunately, the complication is difficult to diagnose, and the signs and symptoms are neither sensitive nor specific given that the presentation may be atypical or delayed [6]. Therefore, if a high index of suspicion is present, we should rely on imaging techniques such as transthoracic echocardiography, CT, or MRI to differentiate whether it is a true or false aneurysm, which, in turn, dictates the treatment modality. Surgical intervention is the most reliable method of treatment according to the literature, but it is worth noting that even with surgical repair, the reported mortality rate is still high up to 7% [7]. Large defects necessitate patch repair; however, smaller defects in the myocardium can be managed with direct primary suture repair [7]. In our case, the patient’s pseudoaneurysm was an incidental finding, yet surgery was indicated to prevent future complications. With simple direct suture repair, the defect was closed and the patient recovered quickly.

## Conclusions

It is crucial to diagnose and treat left ventricular pseudoaneurysms promptly, as any delay in treatment can be fatal, and early diagnosis and intervention are critical to improve patient outcomes. A multidisciplinary approach is also indicated in such complex cases to formulate an efficient management plan. We hope our case enriches the published literature on this rare yet fatal complication and highlights the need for early recognition and adequate surgical treatment to decrease the associated high mortality.
